# Special Issue “Saliva and Oral Diseases”

**DOI:** 10.3390/jcm9061955

**Published:** 2020-06-23

**Authors:** Pia López Jornet

**Affiliations:** Oral Medicine in the Department of Stomatology, Faculty of Dentistry, University of Murcia, 30008 Murcia, Spain; majornet@um.es

The discovery of microbial, immunological or molecular markers in saliva offers unique opportunities, and has caused our view of saliva to change drastically in recent years. Technological developments have made it possible to advance more rapidly and precisely in the diagnosis of diseases—opening a field of research with tremendous potential. In this special issue, The Journal of Clinical Medicine has gathered a panel of experts on saliva research. The different contributions cover a broad spectrum of topics, such as odontology, obesity, inflammation and cancer.

The possibility of monitoring, of seeing how and when a disease process starts and progresses, and of observing the outcomes of treatment based on noninvasive tools such as saliva, is a very desirable goal. One of the most ambitious aims of researchers is to detect salivary biomarkers and use them in clinical practice. Apart from the minimally invasive procedure involved in collecting saliva samples, we have the possibility of compiling lots of data and of exploring intraindividual variations—generating new challenges and opportunities.

In the area of oral diseases, Martina et al. [1] contributed a thorough review on the use of saliva from the perspective of the oral manifestations of skin disease, such as lichen planus, pemphigus, pemphigoid and psoriasis, and also addressed Sjögren’s syndrome and oral cancer.

The working group of the University of Murcia, led by López–Jornet et al. [2], investigated burning mouth syndrome, contributing very interesting data on the determination of analytes in unstimulated saliva implicated in pain, stress and inflammation, and which can offer clues to help manage this complex chronic orofacial pain condition.

Regarding dental implantology, there is much controversy on the risks of peri-implantitis. Papi et al. [3] addressed this issue in an interesting study on saliva, examining the release of metals (titanium) in the development of peri-implantitis.

Overweight people/obesity is a worldwide social problem. Two interesting documents on this topic have been presented from different points of view: (a) Zalewska et al. [4] found overweight and obese adolescents to have an impaired systemic and salivary oxidative status when compared with adolescents of normal weight. (b) In a cross-sectional study, the Finnish research group of Syrjäläinen et al. [5] found that obese people may be susceptible to periodontal disease, though obesity was not associated to alterations in cytokines in saliva. In this regard, salivary cytokine alterations may be explained by a periodontal condition and smoking habit.

The use of saliva opens a range of possibilities for the management of patients with psoriasis. In this regard, biomarkers are explored that can be measured using simple, innocuous, painless and low-cost tests for use in clinical practice. The study [6] of variations in the concentration of TNF-α, IL-2, INF-γ, IL-10, nitric oxide, peroxynitrite, S-nitrosothiols and nitrotyrosine contributes to further our knowledge on the pathogenesis of psoriasis.

Studies on precise, profitable and noninvasive diagnostic methods require important efforts from all researchers. The emerging technologies developed in the last decade have contributed new value to the study of saliva.

Monedeiro et al. [7] used matrix-assisted laser desorption/ionization-time-of-flight mass spectrometry (MALDI-TOF MS) to investigate the influence of silver nanoparticles (AgNP) upon metabolism in bacterial strains. The approaches used may be useful for monitoring and assessing the response to treatment, based on the concentrations of AgNP.

In the field of oncology, we seek to identify molecular alterations and their relation to tumor progression, with a view of developing potential applications to diagnosis, prognosis and response to therapy. In this regard, the Hungarian group of Márton et al. [8] have analyzed the expression of IL-6 and mRNA in samples of saliva from patients with oral squamous cell carcinoma (OSCC), and recorded the differences with respect to the control group. On the other hand, the detection of human papillomavirus (HPV) from saliva (with or without oral rinses) represents a quick and easy noninvasive alternative for oral and oropharyngeal cancer screening in high-risk populations. Rapado–González et al. [9] conducted a meta-analysis providing additional evidence that salivary HPV is associated with oral and oropharyngeal cancer.

Lastly, it is important to create and integrate interdisciplinary research teams capable of advancing knowledge in the field of saliva, since we need to optimize identifying biomarkers and validate the findings in new patients, as well as establish targets for early therapeutic interventions.
